# A Robust and Device-Free System for the Recognition and Classification of Elderly Activities

**DOI:** 10.3390/s16122043

**Published:** 2016-12-01

**Authors:** Fangmin Li, Mohammed Abdulaziz Aide Al-qaness, Yong Zhang, Bihai Zhao, Xidao Luan

**Affiliations:** 1 Department of Mathematics and Computer Science, Changsha University, Changsha 410022, China; lifangmin@whut.edu.cn (F.L.); bihaizhao@163.com (B.Z.); 2 School of Information Engineering, Wuhan University of Technology, Wuhan 407003, China; alqaness@whut.edu.cn (M.A.A.A.); zhang_yong@whut.edu.cn (Y.Z.)

**Keywords:** Wi-Fi, activity recognition, device-free, feature extraction, Principle Component Analysis

## Abstract

Human activity recognition, tracking and classification is an essential trend in assisted living systems that can help support elderly people with their daily activities. Traditional activity recognition approaches depend on vision-based or sensor-based techniques. Nowadays, a novel promising technique has obtained more attention, namely device-free human activity recognition that neither requires the target object to wear or carry a device nor install cameras in a perceived area. The device-free technique for activity recognition uses only the signals of common wireless local area network (WLAN) devices available everywhere. In this paper, we present a novel elderly activities recognition system by leveraging the fluctuation of the wireless signals caused by human motion. We present an efficient method to select the correct data from the Channel State Information (CSI) streams that were neglected in previous approaches. We apply a Principle Component Analysis method that exposes the useful information from raw CSI. Thereafter, Forest Decision (FD) is adopted to classify the proposed activities and has gained a high accuracy rate. Extensive experiments have been conducted in an indoor environment to test the feasibility of the proposed system with a total of five volunteer users. The evaluation shows that the proposed system is applicable and robust to electromagnetic noise.

## 1. Introduction

For decades, human activity recognition has been an important area of human-computer interfaces (HCI). Recent advances in human activity recognition (HAR) and sensing technologies have presented various ideas aiming to enable novel applications in different areas such as healthcare, security, and entertainment [1,2]. A huge amount of literature has been presented in this important search field by using various technologies such as vision-based [3], acoustic-based [4], accelerometer [5], wearable sensors [6], environment installed sensors [7,8], and smartphones [9].

However, such approaches depend on a special device that may be considered as the main drawback of these systems, for instance: a vision-based system requires a camera to monitor human activity, which causes a privacy concern for the target people. Furthermore, the camera is not suitable in some special places such as a “bathroom”. In a bathroom, the activity monitoring is very important in special cases such as elderly fall detection because a bathroom is a potential place for falling. Moreover, the camera cannot traverse through walls. In addition, the camera requires good lighting conditions. The second type of traditional approach is a sensor-based activity recognition system. Such a type is inappropriate for human usage because sometimes elderly people may forget to carry or wear such sensors. Furthermore, all device-based activity recognition systems whether vision-based or sensor-based are not free and require expensive installation, maintenance, and more exhaustive work.

The recent trend in HAR is wireless-based sensing technology or so-called device-free activity recognition that uses only the emitted signals from a wireless transmitter such as wireless local area network (WLAN) 811.b.n.c. Earlier, Woyach et al. [10] proved that the shadowing effect caused by a human subject moving in the line-of-sight path between two communicating wireless nodes can be used for sensing human motion without sensor devices. They supposed that each wireless network is capable of becoming a sensor network. They observed that the motion of bodies in an indoor environment leaves a characteristic footprint on signal strength patterns, which may be leveraged for motion detection. Here, the wireless-based sensing mechanism depends on analysing the collected radio frequency (RF) data from ubiquitous WLAN routers without sensor nodes such as [11,12,13] or RFID tags such as [14,15]. According to this observation, Youssef et al. [16] introduced the concept of passive device-free human tracking system by leveraging the Received Signal Strength Indicator (RSSI) from a ubiquitous Wi-Fi router in indoor environments. According to this phenomenon, many approaches have been presented in various functions such as localization [17], motion detection [18], and human activity recognition [19].

Most recent, Channel State Information (CSI) of the physical layer of the radio system has been used in tracking human motion and activity. CSI has attracted more attention in recent years since CSI can be exported from commodity wireless Network Interface Controller (NIC) [20]. CSI reflects the varying multipath reflection caused by human motion due to its frequency diversity [21]. CSI is more stable and has more information than RSSI because CSI is measured per orthogonal frequency-division multiplexing (OFDM) from each packet, while RSSI is measured by a single value per packet. Therefore, CSI is more robust to the complex environments [22].

Wu et al. [23] presented a CSI-based localization system known as Fila (fine-grained indoor localization) by leveraging the fluctuation of CSI across wireless link subcarriers in the well-known orthogonal frequency diversity multiplexing (OFDM). They extracted CSI from the receiver, and then applied some signal processing techniques in both time and frequency domain and machine algorithms to detect the position of an entity. Thereafter, CSI has been leveraged in various applications such as localizations [24,25], motion detection [26,27], and activity recognition [28,29,30]. WiFall [29] considered the abnormal behavior by applying a local outlier factor based algorithm and classifying the fall action by using a one-class support vector machine (SVM). Wang et al. [30] presented a Wi-Fi based human activity recognition system by leveraging the CSI of Wi-Fi signals, and they also applied an outlier factor algorithm across the CSI stream and applied the SVM to classify human action. However, in [22], only a 1×3 Multiple-Input and Multiple-Output (MIMO) system has been leveraged which may not be enough to track human motion. Moreover, such methods have not extracted the phase information of CSI. They only leveraged the amplitude information. Moreover, such approaches have not presented a typical information selection method. By observing the extracted CSI streams, we noticed that each CSI stream has a different sensitivity to the human motion. Thus, the fluctuation of CSI streams due to human motion is distinctive in each stream. In some streams, the affection of noise is high. Therefore, such streams do not have enough fluctuation.

Unlike previous approaches, in this paper, we extract both amplitude and phase information of CSI. Moreover, we focus on extracting the CSI information in a realistic environment, which means in the existence of different radio device working in the test area at the same time of the testing experiments. The surrounding devices cause real noise to the CSI, and such noise cannot be removed by filtering because such noise affected some CSI streams and distorted the real trend of them. We observed that CSI streams have a distinctive sensitivity to the human motion. In some streams, the affection of human motion is less than others, and the affection of surrounding noise is more than other streams. Therefore, the fluctuation of such streams due to the human motion is not clear. Previous approaches such as [30] extract CSI from each reported stream, and this may cause a false detection. Moreover, in some approaches, such as [29], they calculate the mean value of all streams, and this also causes a false detection in some tests. Instead, we apply Principal Component Analysis (PCA) afcross CSI streams to reduce dimensionality and to obtain the real trend of CSI.

Thereafter, we extract features from both time and frequency domains of principal components of CSI. Finally, our system uses Random Forest classification to recognize implemented activities. Experimentally, we find that the CSI-PCA method promotes the classification accuracy.

Our main contributions can be summarized as follows:

- We present a device-free activity recognition system, which requires only a ubiquitous Wi-Fi router, where the user is free and does not need to wear sensors or be monitored by a camera. The proposed system can classify seven daily activities in line-of-sight (LOS) and none-line-of-sight (NLOS) scenarios.
- Unlike previous CSI-based human activity recognition approaches, we extract both CSI amplitude and phase information, thus providing us with more information.
- We present an efficient CSI-PCA method to reduce dimensionality and to expose the real trend of the CSI stream that is caused by human motion.
- We adopt the Random Forest classification algorithm to classify the proposed activities.
- We present empirical results of various activities during exhaustive experiments conducted in a real-word environment and in different scenarios.

The reminder of this paper is organized as follows. In the next section, we describe the system architecture and methodology. Section 3 shows the experiment setup and the evaluation results. Section 4 discusses the current CSI-based activity recognition method and highlights the limitation and challenges. Lastly, Section 5 concludes the paper.

## 2. System Architecture and Methodology

**Figure 1:**
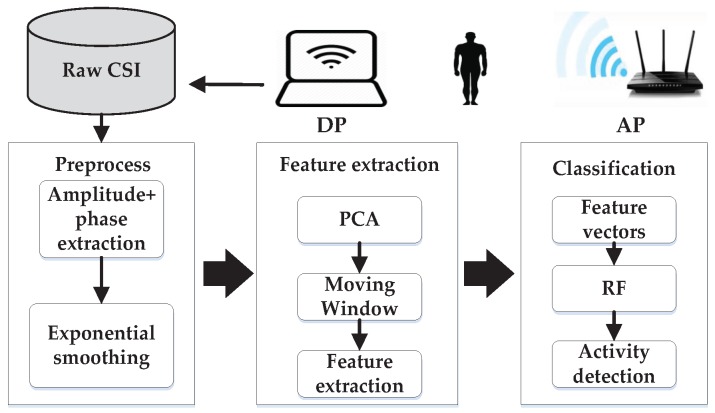
System architecture and work flow.

This section describes the core part of the proposed system. In what follows, we give a detailed breakdown of our system architecture and implementation. One of the key points of elderly activity recognition systems based on CSI is to extract the feature of each motion from filtered raw CSI after applying the PCA algorithm across CSI streams to expose information that we need. Therefore, our novel system consists of the following modules: the CSI Preprocessing module, Feature extraction module and Classification module as shown in Figure 1.

The CSI preprocessing module carries out some basic processes to extract amplitude and phase information that we use. An exponential filter is also implemented to filter out electromagnetic interference caused by 2.4 GHz devices around the testing environment. The main purpose of the PCA is to reduce dimensionality and to expose the real trend of CSI. Since the speed and duration of each motion are different, we can obtain a dynamic time window via the analysis of each motion for extracting features in the latter module. In the last module, we test the classification algorithm on the training and testing sets.

### 2.1. Preprocessing

The proposed system collects radio signals from the fixed access point in the test environment. Let $T_{N}$ denote the number of transmit antennas and $R_{N}$ denote the number of receive antennas. The received signal at the receiver end is expressed as Equation (1):

(1) $$Y_{i}=X_{i}CSI_{i}+N_{i},$$

where *Y* and *X* represent the $T_{N}$ dimensional transmitted signal vector and $R_{N}$ dimensional received signal vector, respectively, in which *i* is the number of OFDM subcarriers and *N* is the noise in each subcarrier. CSI can be represented as Equation (2):

(2) $$CSI_{i,j}=∥CSI_{i,j}∥e^{j∠CSI_{i,j}},$$

where $∥CSI_{i,j}∥$ is the amplitude and $∠CSI_{i,j}$ is the phase, in which *j* is the number of CSI streams. The measurement of CSI depends on the amplitude and phase of CSI at a time point. If we have a number of transmitter antennas $T_{N}$ and receiver antennas $R_{N}$, the CSI of each time point is measured on 30 OFDM subcarriers reported from Intel Wifi Link IWL 5300 [20]. In this case, the subcarrier number is 30 in each $T_{N}\times R_{N}$ stream. In our system, we use a transmitter with two antennas and a receiver with three antennas; thus, we have 2×3×30. Overall, we have 180 data points that can represent CSI in each time point. Thus, CSI can be described as Equation (3):

(3) $$CSI=\left[\begin{array}{ccc} CSI_{1,1} & ... & CSI_{1,30}\\ : & : & :\\ CSI_{6,1} & ... & CSI_{6,30} \end{array}\right]+N.$$

**Figure 2:**
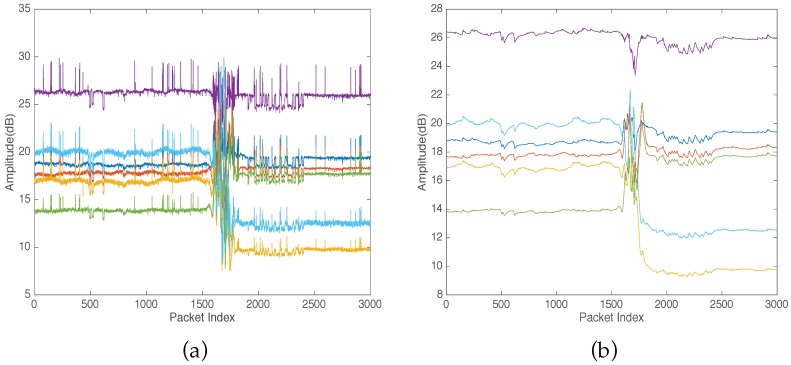
Extracted Channel State Information (CSI) streams from fall experiment. (**a**) Original CSI streams; (**b**) CSI streams after exponential filter.

Many methods can be applied to represent the collected CSI data. Here, to reduce computational complexity and reduce data dimensions, we use the mean of the 30 subcarriers of each stream as a vector that represents CSI. Thus, in our system, CSI is measured as six streams. Figure 2 plots CSI of a fall experiment; as shown in Figure 2a, the collected data still has noise from the neighboring radio devices. Therefore, we design an exponential filter to remove the noise and obtain the real trends of CSI streams that were caused because of human activity. Figure 2b shows CSI streams after filtering. The figure shows that our filter is effective in terms of filtering out the noise.

### 2.2. Feature Extraction

#### 2.2.1. Principal Component Analysis

The PCA [31] is a useful statistical method leveraged in various forms of statistical analysis such as biomedical signal processing [32], computer graphics and pattern recognition [33]. PCA helps identify patterns in the data and express the data in a way that highlights their similarities or differences. Moreover, PCA can also be used to compress the data by reducing the number of dimensions, without much loss of information.

**Figure 3:**
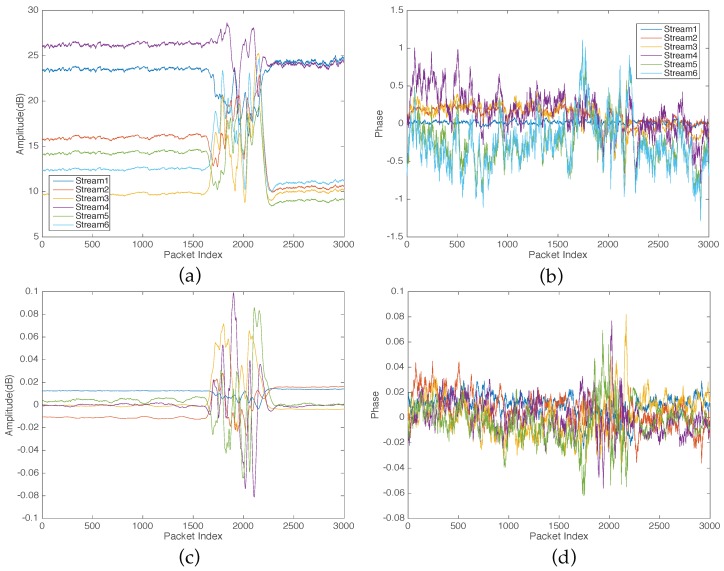
CSI of Walk experiments and Principle Components Analysis (PCA). (**a**) Original CSI amplitude; (**b**) Original CSI phase; (**c**) Principle Components (PCs) of CSI amplitude; (**d**) Principle Components (PCs) of CSI phase.

The fluctuation of CSI measurements caused by human motion occurs across all the streams and subcarriers in the wireless channel. The different streams have a distinctive sensitivity to the fluctuation of CSI due to human motion as shown in Figure 2. To capture the dominant fluctuation of CSI caused by human activity motion, we apply PCA across all the streams of the raw CSI that were collected from IWL 5300 NIC and filtered out by the exponential filter. Therefore, for reported CSI streams, we perform PCA to obtain *p* principle components, as shown in Figure 3c,d. As shown in Figure 3c,d, the PCs discover the CSI fluctuation pattern due to human motion clearer than the original raw CSI (Figure 3a,b). Thereafter, we use the five principal components of both CSI amplitude and phase as shown in Figure 3 for feature extraction.

#### 2.2.2. Feature Selection

We apply a moving window across CSI principal components (CSI-PCs) to calculate the following features: (1) normalized standard deviation (STD); (2) median absolute deviation (MAD); (3) interquartile range (IR); (4) signal entropy; and (5) duration of human motion. Features (1), (2), (3) expose the differences of different motions in time domain distribution of CSI. Signal entropy reflects different amounts of information in different motions. Duration is an important and useful feature in our system, since it varies widely over different actions.

### 2.3. Classification

We build our prototype with the Random Forest classification method. Random Forest was invented by Breiman [34], and can be defined as an ensemble of decision trees and is based on ensemble learning methods for classification and regression. The Random Forest classifier consists of a collection of single classification trees, in which each tree grows by randomly drawing samples, with replacement, from the training set. Random Forest (RF) improves the classification performance of a single-tree classifier by combining the bootstrap aggregating (bagging) method and randomization in the selection of partitioning data nodes in the construction of decision trees. In [35], the authors proposed a classification methodology to recognize human motion using acceleration data, different classes of motions, such as driving a car, being in a train, and walking, by comparing different machine learning techniques (Random Forests, SVM and Naive Bayes). The authors showed that the Random Forest algorithm provides the highest average accuracy outperforming the SVMs and the Naive Bayes. Random Forest classifies a new activity from an input feature vector by putting it onto each of the trees in the forest. Each tree gives a classification decision by voting for that class. Then, the forest chooses the classification having the most votes (over all the trees in the forest). Here, we adopt RF to classify human activities and we find that RF outperforms the popular classification algorithms as described in Section 4.

## 3. Evaluation

### 3.1. Experiments Setup

We implement all the experiments in a laboratory building with a lot of devices working at 2.4 GHz. Multipath signals are very rich in this building. During the experiments, we set the period of each experiment to 30 s, and the motion happens beginning about 15 s. The packet rate is 100 Hz, so that we can get enough information of each motion. In order to detect fall from daily life motions, we test seven motions: walk, sit down, lie, stand up, squat down (i.e., pick up something), fall, and crawl.

We implemented our system using off-the-shelf devices. The system consists of two devices: one WiFi access point (AP) which serves as the transmitter and has three transmitter antennas, and one HP laptop equipped with an Intel WiFi link IWL 5300 802.11n chipset, which serves as a detection point (DP) and has three receiving antennas. The laptop installed Ubuntu 14.04 with a modified Intel NIC driver.

The software that was used in our experiments is the open source CSI-Tools presented by Halperin et al. [14]. After data collection, Matlab software was used to analyze the collected data as described in methodology section.

We conducted experiments with five different volunteer users aged between 22 to 33 years. Each volunteer user was asked to implement the activities described in Table 1 individually.

**Figure 4:**
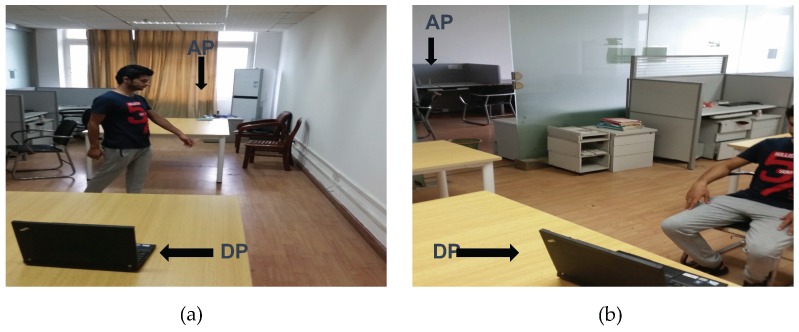
Experiment environment and settings. (**a**) Line-of-sight (LOS) scenario; (**b**) Non-line-of-sight (NLOS) scenario.

The proposed activities implemented in two scenarios:

1. LOS scenario: in this scenario, the target user, the detection point (DP) and the access point (AP) are placed in the same room as shown in Figure 4a.
2. NLOS scenario: in this scenario, the access point (AP) is fixed in a room where the target user and the detection point (DP) are placed in another room as shown in Figure 4b.

### 3.2. Results

#### 3.2.1. Evaluation Metrics

We collected 500 samples of each activity from five users divided into five groups. In each group, 100 samples of each activity were collected from five users as 20 samples of each activity per one user as described in Table 2.

Thereafter, we build our classifier in each group with 100 samples of each activity, and we measure the five-cross validation accuracy. Each user implements the intended activity individually close to the detection point as shown in Figure 4. It is worth mentioning that, in each group, the users must implement the proposed activities in a fixed place because CSI values in each location are different.

**Figure 5:**
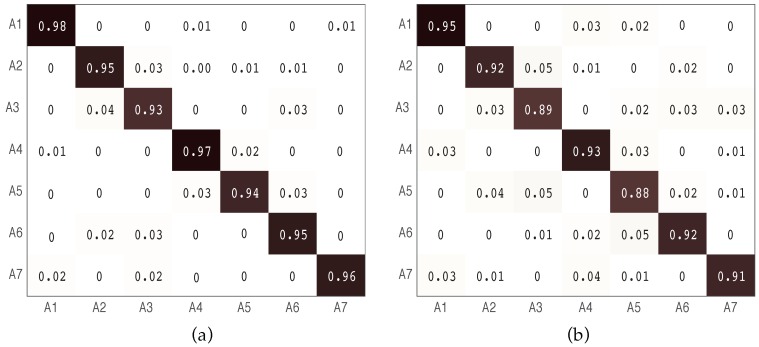
The confusion matrices in both LOS and NLOS scenarios. (**a**) The confusion matrix in an LOS scenario; (**b**) the confusion matrix in an NLOS scenario.

**Figure 6:**
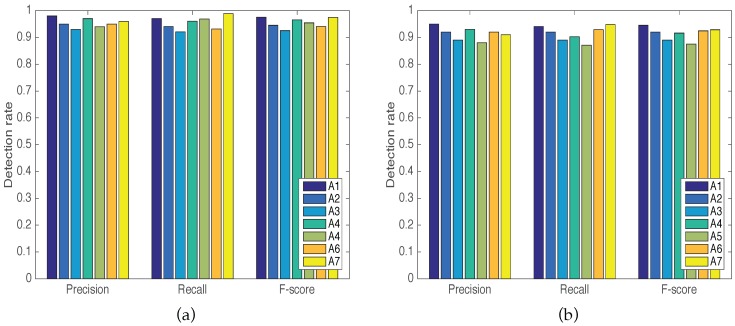
Precision, recall, and F-measure results. (**a**) The results of precision, recall, and F-measure in an LOS scenario; and (**b**) the results of precision, recall, and F-measure in an NLOS scenario.

Figure 5 shows the confusion matrix of the experiment results in both LOS and NLOS scenarios. Each raw represents an actual class, where each column represents a predicted class. From confusion matrices shown in Figure 6, the averaged accuracy is 95.43% in LOS and 91.2% in NLOS.

Moreover, to evaluate the performance of the proposed system, precision, recall, and F1 score are used to analyze results of the experiments. Precision is the positive predictive value, recall is the sensitivity, and F1 score or the F-measure is the weighted average of both the precision and the recall. These three mathematics rules are used as expressed in the following expressions:

(4) $$Precision=\frac{TP}{TP+FP},$$

(5) $$Recall=\frac{TP}{TP+FN},$$

(6) $$F1=2\times \frac{precision\times recall}{precision+recall},$$

where TP, FP, and FN are the true positive, false positive, and false negative, respectively. Figure 6 shows the result of the precision, recall and F1 of the proposed seven activities in LOS and NLOS scenarios.

#### 3.2.2. Validity of PCA Method

**Figure 7:**
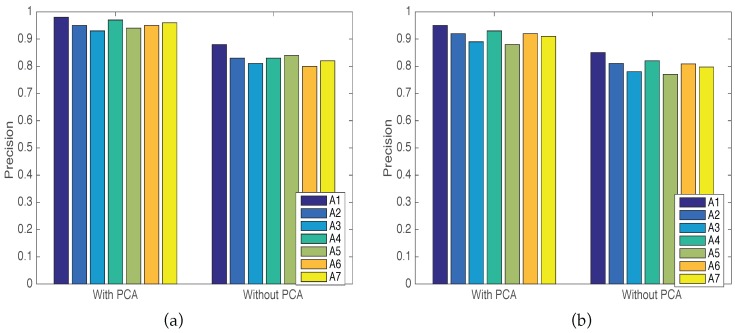
Comparison of system precision with PCA method and without PCA. (**a**) Comparison results in the LOS scenario; (**b**) Comparison results in the NLOS scenario.

To show the validity of the Principal Component Analysis (PCA) method, we measure the precision of each implemented activity in both LOS and NLOS scenarios with PCA and without applying the PCA method. Figure 7 shows that the system precision decreased in cases without the PCA algorithm. The distinctive sensitivity of the CSI streams leads to false detection in some tests, so the precision decreased as shown in the figure.

#### 3.2.3. RSSI vs. CSI

It is very important to test the difference between both device-free sensing metrics, namely RSSI and CSI, since RSSI-based device-free activity recognition systems were adopted earlier. In order to also prove the superiority of CSI over RSSI, we experimentally compare the proposed seven activities in LOS scenarios based on RSSI and CSI.

**Figure 8:**
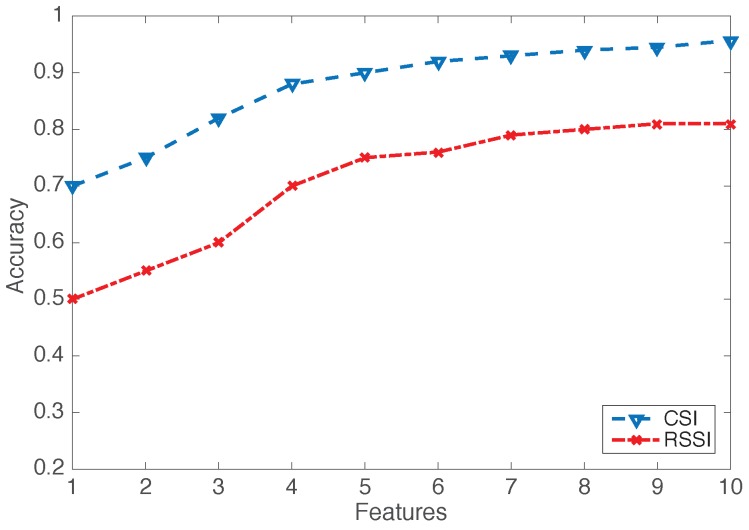
Channel State Information (CSI) vs. Received Signal Strength Indicator (RSSI) in an LOS scenario.

Figure 8 shows the clear superiority of CSI over RSSI. As described above, RSSI is coarse grained, which is measured by a packet index, where, in CSI measured by OFDM subcarriers, the information in CSI is more than RSSI, so the environment noise may distort some streams, but CSI still has real information in other streams.

The main advantage of RSSI over CSI is that RSSI is available in wireless devices, whereas CSI still obtained only with specific NIC cards such as IWL 5300.

#### 3.2.4. Classification Algorithm Comparison

We compare Random Forest with popular classification algorithms such as Support Vector Machine (SVM) and Naive Bayesian Classifier (NBC). We test the accuracy of the three classification algorithms of all the collected data in LOS scenarios. Table 3 shows the average accuracy of each implemented activity. As shown in the table, the RF algorithm outperforms SVM and NBC algorithms.

Overall results showed the validity and stability of our proposed system. The second stage of this system is to extend it to classify all potential human activities and to test multiple people activities at the same time. An important issue to be addressed in the future work is to test human activity in case of interference of moving objects in the test area.

## 4. Discussion

In this paper, the proposed human activity recognition system is motivated by a need for decreasing the installation complexity and development costs induced by the traditional device-based activity recognition systems. As described above, the proposed system can recognize seven daily activities by analyzing the channel state information of Wi-Fi signals with only a ubiquitous Wi-Fi router as an access point and a laptop as a detection point. As mentioned in the results section, the system has gained a high accuracy rate that justified the validity and stability of the proposed methodology.

The device-free CSI-based sensing technique is a promising technique, which may go far in the future. However, there are three main challenges for further study. Here, we highlight such challenges as the following:

1. The presence of moving objects in the test area. This is a complex challenge since CSI is very sensitive to an object’s motion, especially if the moving object is a big object like a big pet (i.e., dog).
2. Since a device-free concept means no need for using special devices in the monitored area or on the perceived object body, it is a challenge to detect two or more human actions at the same time. The solution of such a problem depends on more improvement to the wireless sensing technique.
3. CSI outperforms RSS in tracking human motion and activity classification since CSI has a unique capability to eliminate multipath effects and is more robust to environmental changes due to the fact that CSI has more information than RSS since CSI measured per OFDM subcarrier while RSS measured per packet. However, CSI can be obtained only on specific hardware such as IWL 5300 NICs. Thus, it is not appropriate to be utilized in many devices such as tablets and smart mobile devices. Such a limitation needs more development in receiver antenna hardware.

By solving such challenges, the device-free wireless-based sensing technology will be generalized and be available to track human motion and activity. We plan to solve the first and second challenges in our next stage of study, whereas the third challenge needs a manufacturing solution.

## 5. Conclusions

In this paper, we have presented a device-free activity recognition system using the CSI of Wi-Fi signals. We have extracted the amplitude and phase information from the CSI of Wi-Fi signals. We have designed a low-pass filter to remove the electromagnetic noise to obtain the real trend of CSI caused by human motion. To reduce CSI dimensionality and to choose useful information across all CSI streams, we have used the PCA algorithm, and used PC vectors to be CSI representatives. Thereafter, we have extracted features from PCs that were labeled as inputs to the classification algorithm. The Random Forest algorithm has been adopted to classify the proposed activities. We have experimentally conducted our system in two different scenarios with several volunteers. The results have verified the validity of our system in both the LOS and NLOS scenarios as 95.43% and 91.4%, respectively. 

## Figures and Tables

**Table 1:** The proposed activity description.

Activity No.	Activity
A1	Walk
A2	Sit down
A3	Lie
A4	Stand up
A5	Squat down
A6	Fall
A7	Crawl

**Table 2:** Collected samples of each activity in each scenario.

Scenario	Collected Samples	No. of Groups	Samples per Group	No. of Samples per User in Each Group
Scenario 1	500	5	100	20
Scenario 2	500	5	100	20

**Table 3:** Accuracy of different classification algorithms in an line-of-sight (LOS) scenario.

Activity	RF	SVM	NBC
A 1	0.98	0.95	0.90
A 2	0.95	0.91	0.82
A 3	0.93	0.88	0.79
A 4	0.97	0.92	0.83
A 5	0.94	0.86	0.85
A 6	0.95	0.93	0.80
A 7	0.96	0.89	0.84
